# Alkaline Water Electrolysis for Green Hydrogen Production

**DOI:** 10.1021/acs.accounts.3c00709

**Published:** 2024-02-09

**Authors:** Harun Tüysüz

**Affiliations:** Department of Heterogeneous Catalysis and Sustainable Energy, Max-Planck-Institut für Kohlenforschung, Kaiser-Wilhelm- Platz 1, 45470 Mülheim an der Ruhr, Germany

## Abstract

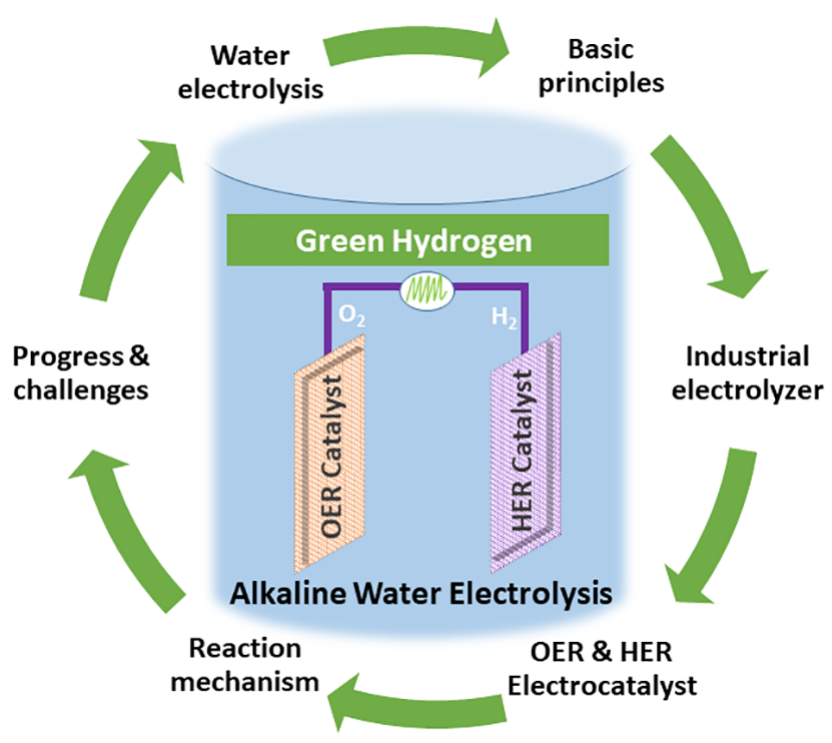

The global energy landscape is undergoing significant change. Hydrogen is seen as the energy carrier of the future and will be a key element in the development of more sustainable industry and society. However, hydrogen is currently produced mainly from fossil fuels, and this needs to change. Alkaline water electrolysis with advanced technology has the most significant potential for this transition to produce large-scale green hydrogen by utilizing renewable energy. The assembly of industrial electrolyzer plants is more complex on a larger scale, but it follows a basic working principle, which involves two half-cells of anode and cathode sites where the oxygen evolution reaction (OER) and hydrogen evolution reaction (HER) occur. Out of the two reactions, the OER is more challenging both thermodynamically and kinetically. Besides having access to renewable electricity, developing durable and abundant electrocatalysts for the OER remains a challenge in large-scale alkaline water electrolysis. Among different physicochemical properties, the electrocatalyst surface and its interaction with water and reaction intermediates, as well as formed molecular hydrogen and oxygen, play an essential role in the catalytic performance and the reaction mechanism. In particular, the binding strengths between the catalyst surface and intermediates determine the rate-limiting step and electrocatalytic performance.

This Account gives some insights into the status of the hydrogen economy and basic principles of alkaline water electrolysis by covering its fundamentals as well as industrial developments. Further, the HER and OER reaction mechanisms of alkaline water electrolysis and selected electrocatalyst progress for both half-reactions are briefly discussed. The Adsorbate Evolution Mechanism and the Lattice Oxygen Mechanism for the OER are explained with specific references. This Account also deliberates on the author’s selected contributions to the development of transition metal-based electrocatalysts for alkaline water electrolysis with an emphasis on OER. The focus is particularly given to the enhancement of intrinsic activity, the role of e_g_-filling, phase segregation, and defect structure of cobalt-based electrocatalysts for OER. Structural modification and phase transformation of the cobalt oxide electrocatalyst under working conditions are further deliberated. In addition, the creation of new active surface species and the activation of cobalt- and nickel-based electrocatalysts through iron uptake from the alkaline electrolyte are discussed. In the end, this Account provides a brief overview of challenges related to large-scale production and utilization of green hydrogen.

## Key References

- BudiyantoE.; SalamonS.; WangY.; WendeH.; TüysüzH.Phase Segregation in Cobalt Iron Oxide Nanowires toward Enhanced Oxygen Evolution Reaction Activity. JACS Au2022, 2, 697–71035373196 10.1021/jacsau.1c00561PMC8970005.^1^*This article reveals the role of phase segregation and boundaries in cobalt–iron oxide for OER. The in situ electrochemical Raman spectroscopy captures the phase transformation from CoO to the defective Co_3_O_4_ spinel structure during OER*.
- YuM. Q.; MoonG. H.; CastilloR. G.; DeBeerS.; WeidenthalerC.; TüysüzH.Dual Role of Silver Moieties Coupled with Ordered Mesoporous Cobalt Oxide towards Electrocatalytic Oxygen Evolution Reaction. Angew. Chem., Int. Ed.2020, 59, 16544–1655210.1002/anie.202003801PMC754046532537829.^2^*This article demonstrates the dual effect of silver species on the OER performance of mesostructured cobalt oxide. Metallic silver particles enhance the conductivity of materials, while well-dispersed silver oxide clusters endow cobalt oxide with the capability of iron uptake from the alkaline electrolyte.*
- YuM. Q.; WeidenthalerC.; WangY.; BudiyantoE.; SahinE. O.; ChenM. M.; DeBeerS.; RudigerO.; TüysüzH.Surface Boron Modulation on Cobalt Oxide Nanocrystals for Electrochemical Oxygen Evolution Reaction. Angew. Chem., Int. Ed.2022, 61, e20221154310.1002/anie.202211543PMC982636536001016.^3^*This article presents a facile synthetic methodology for the preparation of mixed boron–cobalt oxide structures. The presence of boron oxide was found to prevent the sintering of cobalt oxide nanoparticles at higher temperatures and improve their OER performance.*
- YuM. Q.; LiG. W.; FuC. G.; LiuE. K.; MannaK.; BudiyantoE.; YangQ.; FelserC.; TüysüzH.Tunable e_g_ Orbital Occupancy in Heusler Compounds for Oxygen Evolution Reaction. Angew. Chem., Int. Ed.2021, 60, 5800–580510.1002/anie.202013610PMC798672933300643.^4^*This research article shows the potential of Heusler compounds as a new class of OER electrocatalysts whereby e_g_ orbital occupancy was found to significantly influence the OER activity of the compounds*.

## 1 Introduction

Water electrolysis is one of the main options for converting electrical energy into chemical energy by producing hydrogen. Clean hydrogen is considered the fuel of the future, as it can be used in various applications and sectors, including mobility and transportation, as well as for heat and power generation for households and industries. The global H_2_ demand increased by 3% in 2022 and reached 95 million tons. Almost all H_2_ used in industry is produced from fossil fuel sources, such as natural gas and coal. The hydrogen generation and utilization in 2022 caused about 900 Mt of CO_2_ emissions. The amount of green H_2_ produced by water splitting (0.1% of today’s global H_2_ generation) and blue H_2_ produced from fossil fuel-based sources with carbon capture, utilization, and storage was only about 0.7%.^5^

A recent analysis revealed that blue H_2_ production is not a low-emission process as is believed. Total CO_2_ emissions for blue H_2_ production were calculated to be only 9–12% lower than those for gray H_2_ which is produced via steam reforming of natural gas.^6^ The overall greenhouse gas footprint of blue H_2_ was calculated to be more than 20% higher than that of burning natural gas or coal for direct heat generation.^6^ Synthetic H_2_ produced through water electrolysis using renewable electricity will be the only option to harvest zero-emission clean H_2_ for future sustainable applications.

The global installed capacity of water electrolysis for H_2_ production reached almost 700 MW by the end of 2022, which is an increase of about 20% compared to that in the previous year. In the case of all the planned projects becoming operational, the global electrolysis capacity could reach 175 GW in 2030. This capacity could even increase to 420 GW if early stage projects are included. While this is very impressive progress, about 600 GW of operational electrolysis capacity will be needed by 2030 to reach the net-zero CO_2_ target.^5^ Therefore, the planned and attempted projects need to be scaled up and installed at a much faster pace.

## 2 Alkaline Water Electrolysis (AWE)

### 2.1 Basic Principles

**Figure 1:**
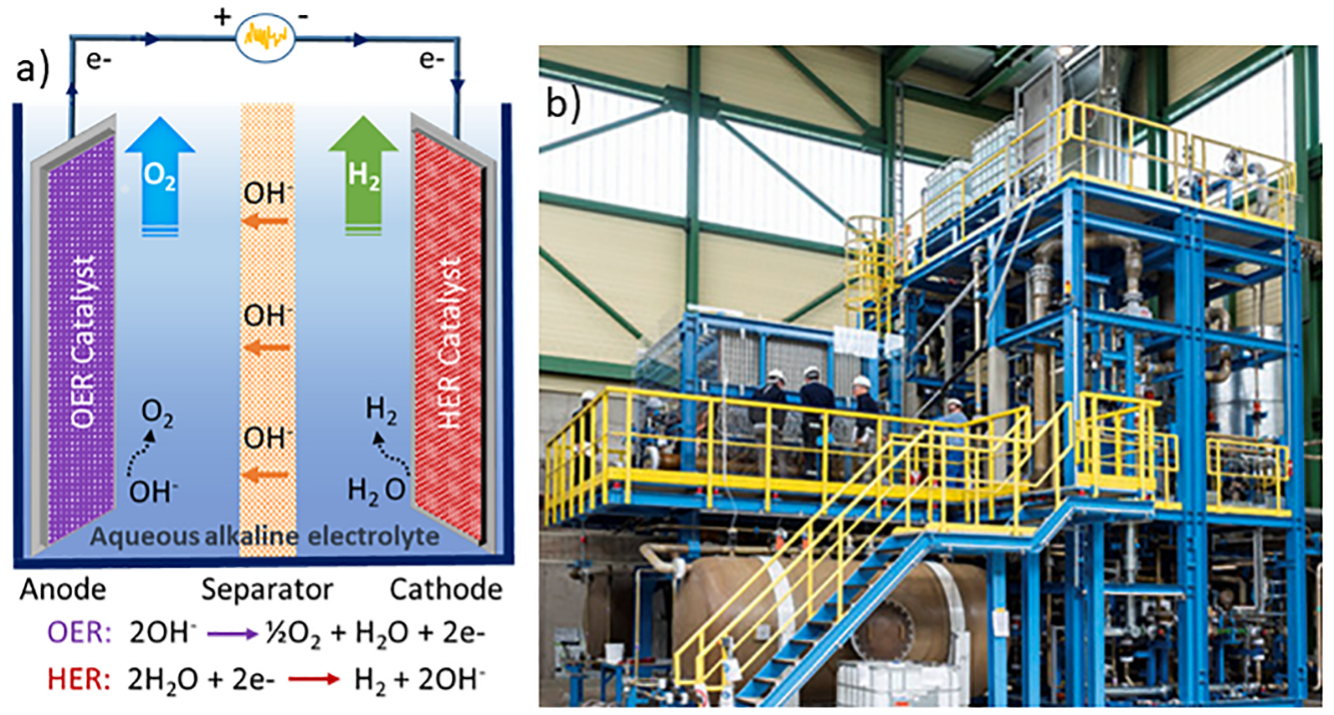
(a) Illustration of a basic AWE cell that consists of an electrical circuit, an electrolyte, a separator, as well as anode and cathode electrodes that are decorated with active catalyst where, respectively, OER and HER take place. (b) Picture of a 2 megawatt (MW) electrolysis plant. Reproduced with permission from ref (9). Copyright 2018 Wiley.

Electrochemical water splitting consists of two half-reactions, namely, the hydrogen evolution reaction (HER) and the oxygen evolution reaction (OER). As illustrated in Figure 1a, these reactions take place at the cathode and anode sites, respectively, and they are preferably separated by an ion-exchange membrane. Among other parameters and indicators (like temperature, pressure, type and concentration of electrolyte, electrode type, and cell configuration), both HER and OER are very sensitive to the pH of the electrolyte, and they proceed in different reaction pathways.^7^ In acidic media, water is oxidized to molecular oxygen, producing protons at the anode. The generated protons are transferred to the cathode and reduced to molecular hydrogen. Under alkaline conditions, as shown in Figure 1a, hydroxide anions, generated by the water at the cathode site, act as electrochemical charge carriers. The oxidation of hydroxyl anions at the anode site results in O_2_ and electrons, which are further used for H_2_ generation at the cathode site. The electrochemical reactions take place at the interface between the electrode surface and the electrolyte, and the gas evolution rate is directly proportional to the flowing current through the electric circuit based on Faraday’s law of electrolysis.^8^ Although the industrial electrolyzer plant assembly is more complicated at a larger scale (Figure 1b), which typically consists of an electrolyzer cell stack, H_2_ and O_2_ separator tanks, cooler, and circulation pump, the basic working concept is based on two half-cells.

Water splitting is thermodynamically an uphill reaction and needs an energy input of Δ*G* = 237.1 kJ/mol; in other words, a thermodynamic potential of 1.23 V is required to drive the water electrolysis.^10^ In reality, a much higher applied voltage, the so-called overpotential (difference between the applied potential and the theoretical value of 1.23 V), is required to overcome several barriers. These include electrical resistance of the circuit, the activation energies of the electrochemical reactions, as well as hurdles related to gas bubbles, that cause additional resistance to ionic transfer and electrochemical reactions, and mass transportation, especially due to the sluggish OER.^7^ To make the process more affordable, the overpotentials for both half-reactions should be lowered. This can be achieved by using electrocatalysts that can reduce the energy input and activation energy, which will be discussed in Section 3.

### 2.2 Industrial Electrolyzer Configuration

Alkaline water electrolysis (AWE) is one of the most practical and advanced technologies with a long history of utilization in the chlor-alkali industry for large-scale H_2_ production. It is typically operated in the temperature range 50–80 °C and at pressures of up to 30 bar. In a classic industrial configuration, the electrocatalyst loaded electrodes made of Raney Ni, Ni (or Fe)-plated steel, or Ni/stainless-steel mesh are immersed in a highly concentrated aqueous KOH solution (typically 20–30 wt %).^11^ A diaphragm made of solid porous oxide (like Zr-based Zirfon Perl UTP 500, Agfa-Gevaert N.V.) that allows transport of OH^–^ between the electrodes is used to separate formed H_2_ and O_2_ gases. This separator provides low gas contamination and a high ionic conductivity, whereby its performance fluctuates depending on the geometrical structure and the composition. The diaphragm is an essential component for the overall performance of the cell, as it ensures the ionic contact between the electrodes and prevents the mixing of the produced gases.^12^

Two different kinds of electrolysis cells are used as a mount, namely, the simple tank cell (unipolar) and the filter-press cell (bipolar) which consists of multiple stacks. The unipolar cells are very compact and have lower ohmic losses while the bipolar cells have more complicated structural designs that require electrolyte circulation and the usage of an external gas separation unit.^11^ Electrolyzer cells can also be categorized as conventional and zero-gap assemblies. The conventional electrolyzer cell assembly is based on maintaining a certain distance between the anode and cathode electrodes, whereas in the case of a zero-gap cell, both electrodes and the separator are pressed together in a layer-by-layer configuration to minimize the distance between the anode and cathode sites. In a zero-gap electrolyzer cell configuration, the ohmic losses that are associated with the voltage drop owing to the relocation of electrons in the electric circuit and movement of ions through the electrolyte and separator can be reduced. But, this brings also some drawbacks with it; reducing the distance causes higher gas bubble accumulation between both electrodes and a decrease in the conductivity of the electrolyte.^13^ One of the main drawbacks of industrial AWE is that it has to be operated at lower current densities typically in the range of 0.05 to 0.7 A/cm^2^ depending on the cell pressure due to the impermeability behavior of the porous membrane for the gas separation.^13^ The AWE has begun to gain more attention due to the increasing demand and interest in green H_2_. This exponential growth and interest is forcing academia and industry to revisit and improve some of its aspects, especially the development of a low-cost and stable electrocatalyst.

## 3 Electrocatalyst Development for Alkaline Water Electrolysis

The activity of the electrocatalyst depends on several physicochemical properties of the material, including the composition, conductivity, electronic and crystal structures, morphology and textural parameters, as well as the preparation method, grain boundaries, surface structure, and the presence of defects.^7,14^ The performance of the electrocatalyst can be boosted either by increasing the number of active sites on a given electrode, through enhanced loading or altering structural properties to expose more catalytic active sites per gram, or manipulating the intrinsic activity of each active site by keeping the mass constant.^15^ The catalyst materials must fulfill some basic requirements and criteria to be considered for large-scale applications. On the one hand, it should be efficient and provide a high current density at low applied potential, should have good structural durability and stability under operating conditions, and should be cost-effective. On the other hand, the figure of merit of the catalyst should be holistic and take into account other key aspects during the electrocatalyst design including sustainability, criticality (covering supply and geopolitical risks of raw materials), ecology, and recyclability. It is very essential to prioritize sustainability and recyclability in every stage of production, considering the limited resources and depletion of many elements in the near future. It is also worth noting that the electrocatalytic performance of a catalyst is highly dependent on the experimental conditions and measurement techniques. For a more detailed understanding of electrocatalytic properties and performance indicators, as well as a protocol for evaluating the activity and stability of OER catalysts, one can refer to other review articles.^7,15,16^

### 3.1 Electrocatalysts and Reaction Mechanism for the HER

**Figure 2:**
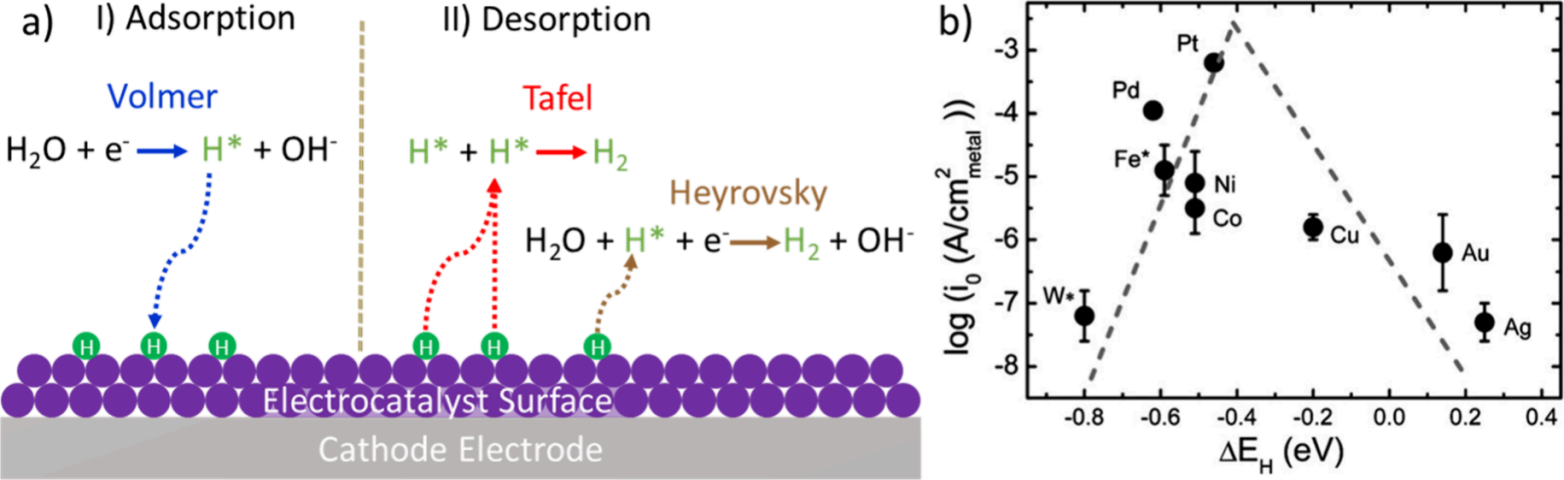
(a) Illustration of alkaline HER consisting of adsorption (Volmer) and desorption (Tafel or Heyrovsky) steps and (b) volcano-shaped curve of the activity of typical HER electrocatalyst in alkaline media, in which exchange current densities (log(*i*_0_)) on monometallic surfaces are plotted as a function of the calculated hydrogen binding energy. Panel b is reproduced with permission from ref (18). Copyright 2013 Royal Society of Chemistry.

The catalyst surface and its interaction with water and reaction intermediates, as well as the formed molecular hydrogen, play an essential role in the catalytic performance and the HER mechanism. The two-electron transfer HER mechanism over a catalyst in alkaline media is based on the cleavage of the O–H bond of water, adsorption of hydrogen species on the electrode surface, formation of the H–H bond, and finally release of molecular H_2_. As shown in Figure 2a, the HER starts with the Volmer step, where H_2_O is reduced and the generated atomic hydrogen is bonded to the surface of the electrocatalyst (donated as H*). This is followed by combination with another hydrogen atom and desorption of H_2_, which might take place either via the Tafel reaction (combination of two adsorbed hydrogen species, H*) or the Heyrovsky pathway (involvement of another hydrogen from the dissociation of the H_2_O molecule), as shown in Figure 2a.^17^

The hydrogen adsorption/desorption steps and the strength of the metal–hydrogen (M–H) bond determine the HER kinetics. According to Sabatier’s principle, the M–H bond should be neither too strong nor too weak to form reaction intermediates and molecular hydrogen.^19^ Hydrogen binding energy is a very essential indicator of the activity of the electrocatalyst, and it is commonly accepted as the ultimate performance descriptor. Exchange currents as a function of the M–H bond strengths show a volcano-shaped curve, which was described by Trasatti in the early 1970s using the experimental data for H_2_ evolution in acidic media.^20^ Nørskov et al. established modern volcano plots by using adsorption energies of electrochemical reaction intermediates, calculated by density functional theory (DFT), as a function of the electrode potential.^19^ As seen in the volcano-shaped curve in Figure 2b, the Pt electrocatalyst shows the highest current density with the optimized hydrogen binding energy for HER in alkaline electrolyte,^18^ which is comparable to the case in acidic media described by Trasatti.^20^

Although this correlation is very beneficial to predict the activity, in reality, the correlation between the hydrogen adsorption energy and the performance of the electrocatalyst for the HER is more complicated. Most metals (especially transition metals) are easily oxidized in alkaline electrolyte, and their surface is covered by oxide layers. Thus, hydrogen atoms would not have direct contact with the metal. This causes a shift in the position of the catalyst in the volcano plot. Further, the reaction kinetics can also be affected by the adsorption of other species (like OH^–^), which can block the active sites and change the energetic states of the adsorbed hydrogen species.^21^

The platinum group metals are well-known as HER electrocatalysts, with Pt being commonly used as a benchmark electrocatalyst for both the alkaline and the acidic media.^22^ However, the high cost and limited resources of Pt necessitate the search for alternative electrocatalysts. Ni-based electrodes are also widely used as cathodes for H_2_ generation in industrial AWE. However, a pristine Ni electrode undergoes structural changes under the harsh conditions of electrolysis. This results in modification of the electrode surface and the formation of other species such as nickel hydride, which leads to rapid deactivation.^23^ A Raney-nickel alloy that is made from Ni–Al has been implemented as an alternative HER catalyst with improved stability.^24^ Among a range of Ni-based binary alloys, the Ni–Mo alloy produced via electrodeposition on steel strips was reported to show very good activity with an overpotential of around 180 mV at 300 mA/cm^2^ for over 1500 h of continuous electrolysis in 6 M KOH.^25^

**Figure 3:**
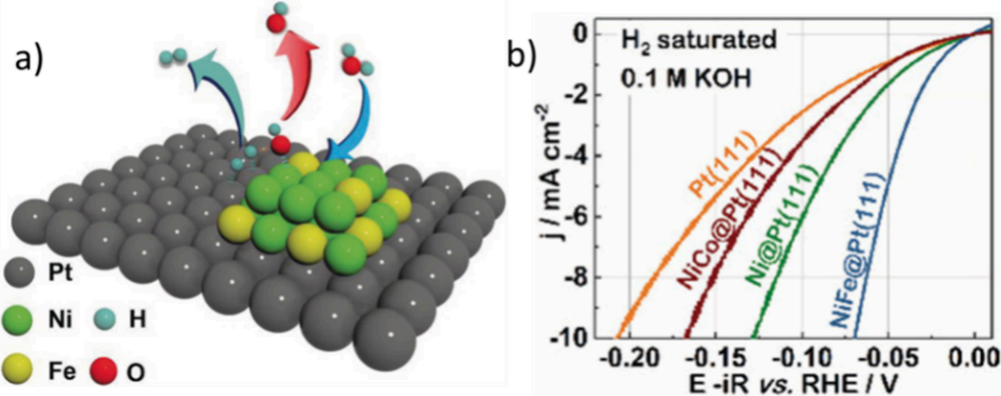
(a) Proposed mechanism for HER over a Ni and Fe metal hydroxide cluster decorated Pt(111) electrode. The Ni–Fe cluster is proposed to improve the initial water dissociation step (blue arrow), and Pt stimulates the adsorption of hydrogen species and H_2_ gas evolution (jade arrow). (b) Polarization curves for metal hydroxide clusters deposited on a Pt(111) single crystal in 0.1 M KOH. Reproduced with permission from ref (27). Copyright 2020 Wiley.

Deposition of nanosized Ni(OH)_2_ clusters on the Pt electrode surface was also found to increase the HER activity by a factor of 8, whereby the edges of the Ni(OH)_2_ clusters could promote the dissociation of water and the generation of hydrogen intermediates.^26^ A similar observation was made with the surface decoration of a Pt(111) single crystal with Ni–Fe and Ni–Co hydroxide clusters (Figure 3a,b). These clusters facilitated water dissociation, and the overpotential required to reach a current density of 10 mA/cm^2^ was reduced to about 70 mV in the 0.1 M KOH electrolyte. The addition of Fe was proposed to help Ni clusters to dissociate water whereby the proposal was supported by the increased *OH binding energies of Ni–Fe clusters.^27^ A nickel oxide/nickel heterostructure supported on carbon nanotubes was reported to have HER activity as good as the commercial Pt/C catalyst.^28^ The high performance of the catalyst was attributed to the NiO/Ni interfaces in the heterostructure, where the NiO and Ni sites could serve as adsorption sites for OH^–^ (produced by H_2_O dissociation) and hydrogen, respectively. A similar synergy could also be observed for hybrid cobalt–cobalt oxide embedded on N-doped carbon, which could be used as both cathode and anode materials for AWE.^29^ A more comprehensive review of HER electrocatalyst development for AWE can be found in the recent review article by Lim.^30^

### 3.2 Electrocatalysts and Reaction Mechanism for the OER

**Figure 4:**
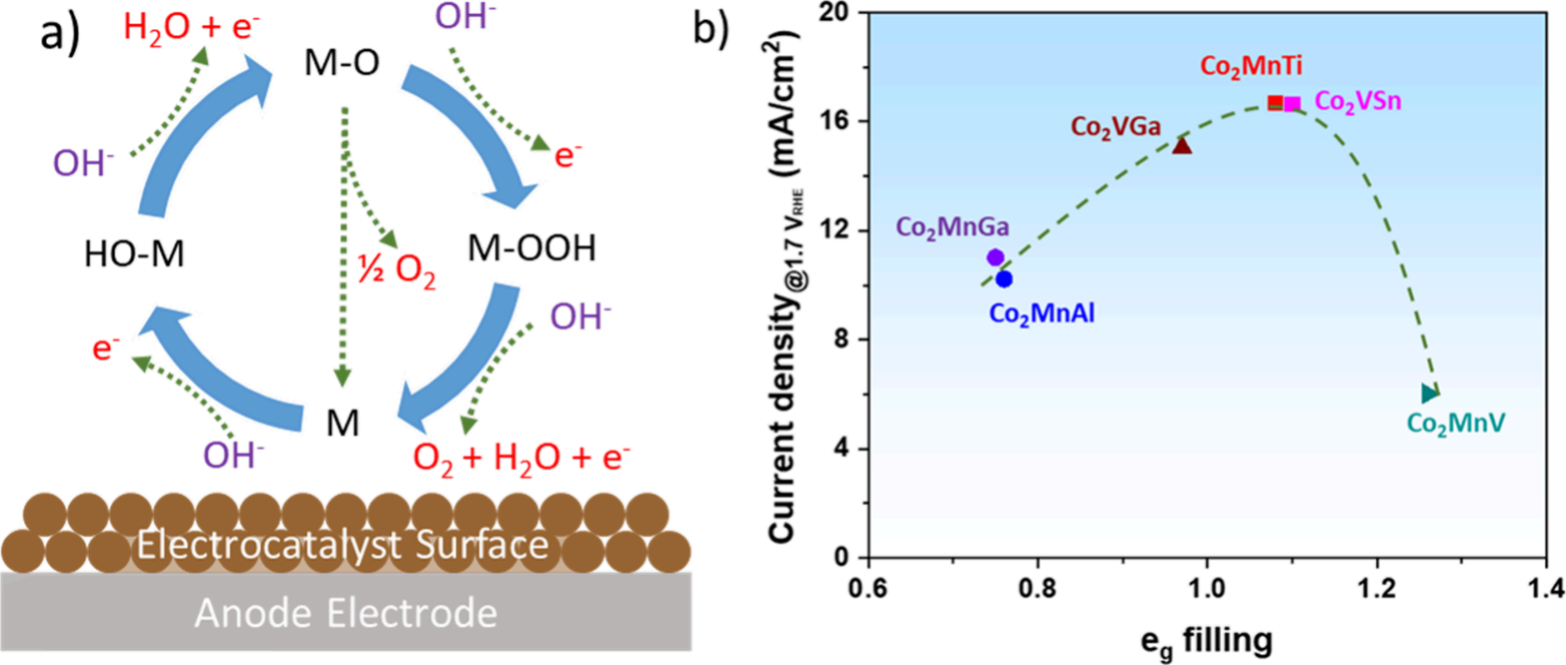
(a) Sketch of the OER mechanism over a catalyst surface and (b) volcano-shaped curve of the OER activity of cobalt-based Heusler compounds, defined by the current density at 1.7 V vs RHE against the occupancy of the e_g_ electron of cobalt. Panel b reproduced with permission from ref (4). Copyright 2021 Wiley.

The OER mechanism is more complicated, since it has several intermediate states with different activation steps that affect its reaction rate. The Adsorbate Evolution Mechanism (AEM) is the most widely accepted mechanism. As shown in Figure 4a, the first step of the OER in alkaline media is the adsorption of hydroxide ion (OH^–^) on the active site of the electrocatalyst (denoted as M), the formation of the M–OH intermediate, and the release of an electron. Further reaction of the M–OH intermediate with another OH^–^ ion results in the formation of the M–O intermediate and H_2_O, and the release of another electron. Molecular O_2_ can be formed from the M–O intermediate via two different pathways: (i) direct combination of two M–O species; (ii) nucleophilic attack of a OH^–^ ion to M–O, creation of M-OOH intermediate and its further reaction with another OH^–^ ion, and finally conversion to water and molecular O_2_.^14^ The second pathway is the most commonly accepted 4e^–^ transfer reaction mechanism in alkaline media.^31^

The binding strengths between the catalyst surface and OH^–^ ions, intermediates (HO*, O*, and HOO*), and products determine the rate-limiting step and the overall OER efficiency of the electrocatalyst. A correlation between the variation of the M–OH bond energy and required overvoltage for the OER in alkaline media was recognized already in 1955.^32^ If the catalyst surface binds too strongly to oxygen, the reaction rate is limited by the formation of the HOO* species. On the other hand, if the catalyst surface binds to oxygen too weakly, the potential is limited by the oxidation of HO* intermediates.^33^ Plotting the performance of electrocatalysts as a function of binding energy results in a volcano-shaped curve whereby oxides like Co_3_O_4_ and RuO_2_ and perovskites like LaNiO_3_ and SrCoO_3_ were shown to display the lowest theoretical overpotentials due to their optimal binding energies.^33^ This could be well correlated with experimental data with the electrocatalytic activity sequence of RuO_2_ > IrO_2_ > Co and Ni containing oxides > Fe, Mn, and Pb containing oxides.^34^ Nonetheless, a solid correlation between theoretical predictions and experimentally measured OER activities on metal surfaces is not straightforward, since the reaction takes place on an oxidized surface in alkaline media.

As an alternative to AEM, the Lattice Oxygen Mechanism (LOM) was proposed initially over the LaNiO_3_ electrocatalyst, which was found to have a lower reaction barrier.^35^ The OER process in both AEM and LOM starts with the hydroxylation of the catalyst’s surface. However, LOM involves direct O–O coupling of oxidation intermediates and the lattice oxygen of the catalyst. Although the reaction intermediates are similar, the LOM differs in the generation of an oxygen vacancy during the evolution of lattice oxygen, linked to the decoupling of a specific proton–electron transfer step. This results in pH-dependent OER kinetics.^36^ The involvement of lattice oxygen in the OER mechanism within the perovskite catalyst has also been verified by using electrochemical mass spectrometry measurements of ^18^O-labeled perovskites.^37^ It has been shown that the OER mechanism can be switched from the AEM to the LOM by substitution of catalytically inactive Zn^2+^ into the CoOOH structure.^38^ The addition of the Zn^2+^ ions was found to give rise to oxygen nonbonding states with different local configurations.

Many physicochemical properties influence the OER performance of the materials. Markovic et al. demonstrated that the strength of the OH_ad_–M^*n*+^ interaction within 3d metal hydr(oxy)oxide catalysts dominantly determine the catalytic activity.^39^ The electronic structure, especially the position of the metal d-band center relative to its Fermi level, plays a significant role in the OER intermediates and molecular O_2_ adsorption strengths. As the adsorbate (OH^–^ ion) approaches the catalyst surface, the electrons of the adsorbate interact with the valence s, p, and d bands of the metal and form an intermediate through the metal–adsorbate bond. The stability and reactivity of the metal–adsorbate bond are largely determined by the number of d*-*electrons. Shao-Horn et al. further revealed that the e_g_ filling of surface transition metal cations could affect the binding of OER intermediates to the oxide surface, and consequently the OER performance.^40^ The author’s team has recently demonstrated that a similar OER activity–e_g_ occupancy trend could be also observed for cobalt-based Heusler compounds (Co_2_YZ) in alkaline electrolyte.^4^ As shown in Figure 4b, the e_g_ orbital filling of catalytically active Co sites could be tuned by varying the Y and Z sites of the Co_2_YZ compounds, where higher catalytic performances were obtained for Co_2_MnTi and Co_2_VSn compounds with e_g_ orbital filling approaching unity. The theoretical prediction supported a preferable OER pathway on Heusler compounds via a direct combination of two M–O species. Alteration of the e_g_ orbital filling could modulate the M–O bonding strength and the OER activity of catalysts.

The oxide electrodes, known as Dimensionally Stable Anodes (DSAs), have shaped electrochemical technologies; Trasatti reported superior hydrogen and oxygen evolution performances of the RuO_2_^41^ and IrO_2_^42^ electrodes in the early 1970s and 1980s. IrO_2_ is used as the state-of-the-art OER electrocatalyst; however, due to its high-cost and limited resources, much effort has been devoted to search for alternative OER catalysts, especially based on abundant first-row transition metals like Mn, Fe, Co, and Ni. The following section will elaborate on selected reports regarding Ni-, Fe-, and Co-based oxides, including studies from the author’s lab with the focus of improving the intrinsic properties of electrocatalysts and the role of iron impurity in alkaline electrolyte.

The study by Corrigan in 1987 revealed a distinct synergy between Ni and Fe for OER activity.^43^ Fe impurities introduced from the commercial KOH electrolyte or co-precipitated in thin nickel oxide electrodes were found to have a strong effect on the electrocatalyst performance. Boettcher et al. revealed that the conductivity of Ni(OH)_2_/NiOOH increases at least 30-fold upon co-precipitation with Fe, which originates a partial-charge-transfer activation effect on Ni that results in OER enhancement, analogous to that observed for noble-metal electrode surfaces.^44^ The origin of the OER activity enhancement of mixed Ni–Fe oxyhydroxides (Ni_1–*x*_Fe_*x*_OOH) over their pure Ni and Fe parent compounds has been also associated with the unusually short Fe–O bond distance, induced by edge-sharing with the surrounding [NiO_6_] octahedron, that results in optimal adsorption energies of OER intermediates and low overpotentials at Fe sites.^45^ A systematic study by the author’s team revealed the distinct effect of composition and the role of the Ni/Fe ratio in NiFe oxides for the OER.^46^ A series of Ni–Fe oxide nanoparticles with an average particle size of about 8 nm and similar textural parameters could be prepared using the pore confinement of the tea leaves as a template. All of the Ni-containing samples were observed to undergo an activation process during electrochemical cycling due to the Fe impurity uptake from the KOH electrolyte.

**Figure 5:**
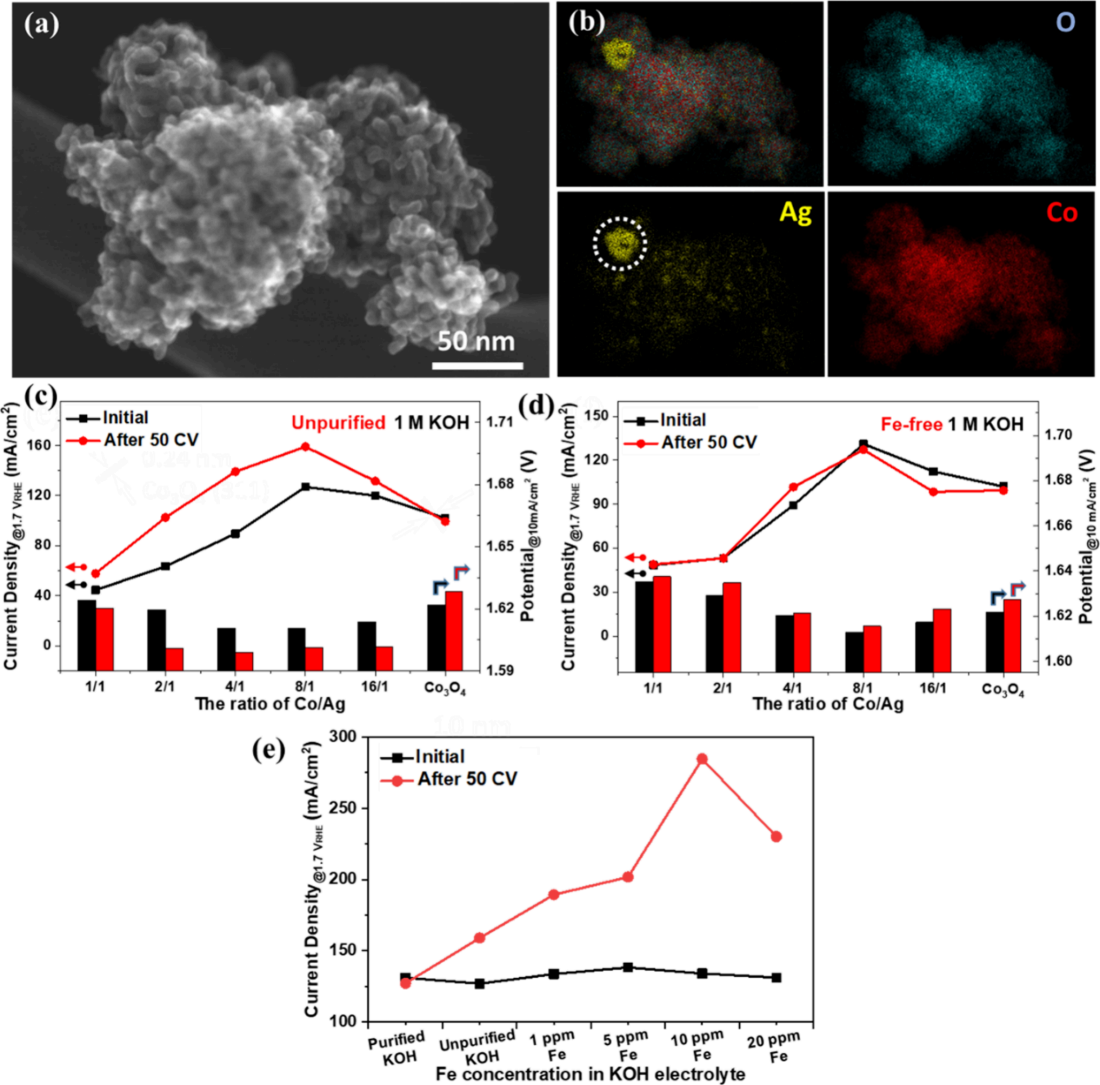
SEM image (a) and the corresponding elemental mapping images (b) of selected silver–cobalt composite oxide. Current density at 1.7 V_RHE_ and applied potential at 10 mA cm^–2^ before and after 50 CV for pristine Co_3_O_4_ and Ag–Co oxides in a 1 M KOH electrolyte with trace Fe impurities (c) and Fe-free 1 M KOH after purification (d). Current density at 1.7 V_RHE_ of selected Co_8_Ag oxides in purified 1 M KOH with varying Fe concentration with the controlled addition of Fe (e). Reproduced with permission from ref (2). Copyright 2020 Wiley.

Although crystalline cobalt oxide spinel gets deactivated upon electrochemical cycling in KOH electrolyte, its combination with nickel has been shown to provide the ability of iron uptake from the KOH electrode and boost OER activity.^47,48^ This kind of activation could be likewise achieved when silver moieties (metallic Ag and silver oxide) were coupled with a crystalline cobalt oxide structure (Figure 5a,b). The author’s team verified that combining silver with ordered mesoporous cobalt oxide structure through the nanocasting route has a dual effect on its OER activity. The incorporation of metallic Ag was found to enhance the conductivity of the oxide, while silver oxide species resulted in the Fe-induced activation of the electrocatalyst.^2^ As seen in Figure 5c, although pristine Co_3_O_4_ showed some deactivation, all Ag-containing samples became activated after 50 cyclic voltammetry (CV) experiments, and the required potential to reach 10 mA/cm^2^ dropped in 1 M KOH with iron impurities.

The activation was not observed when commercial KOH was purified and used as the electrolyte (Figure 5d). When a controlled amount of iron was added to the purified KOH electrolyte, the selected most active cobalt–silver oxide electrocatalyst underwent an activation process again, whereas the presence of 10 ppm of iron impurity resulted in the highest current density, almost reaching 300 mA/cm^2^ at 1.7 V_RHE_, as seen in Figure 5e.

In contrast to its crystalline counterpart, we have verified that amorphous cobalt oxyhydroxide, prepared by the electrochemical deposition method is also capable of iron-uptake from KOH electrolyte.^49^ Overall, the iron-uptake capability is a distinct advantage for the regeneration of catalysts and the creation of more active catalytic centers. However, other impurities such as carbonate and organic species in highly concentrated alkaline electrolytes can also be deposited on Co_3_O_4_, affecting its surface structure and OER performance.^50^

**Figure 6:**
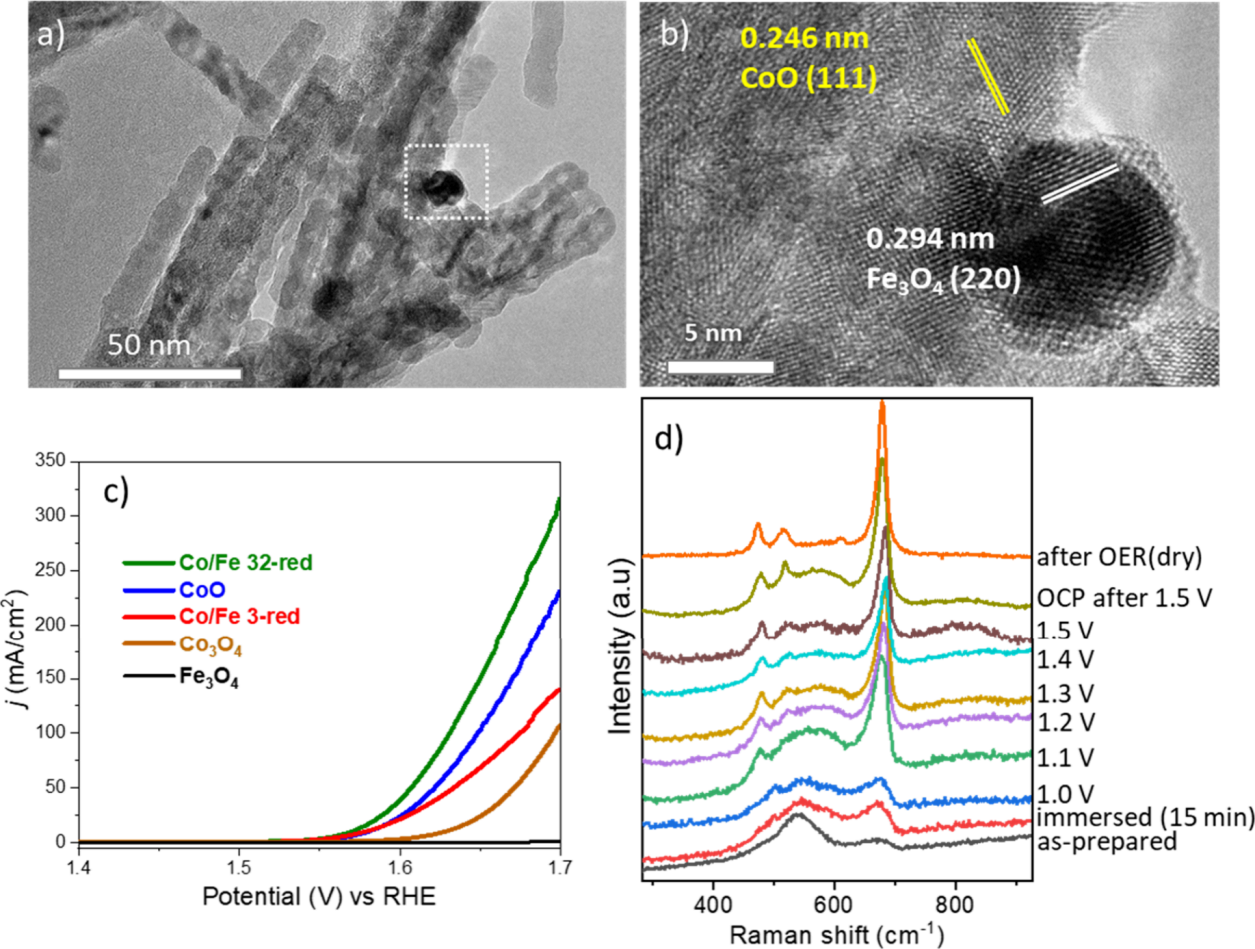
(a, b) TEM and HR-TEM images of reduced, iron oxide nanoparticle supported CoO nanowires, (c) LSV curves of reduced CoO and CoFe oxides with Co/Fe ratios of 32 and 3 (denoted as Co/Fe 32-red and Co/Fe 3-red, respectively), pristine Co_3_O_4_, and Fe_3_O_4_ in 1 M KOH electrolyte. *In situ* electrochemical Raman spectra of the Co/Fe 32-red sample, whereby transformation of CoO into Co_3_O_4_ at higher applied potential could be monitored. Reproduced with permission from ref (1). Copyright 2022 American Chemical Society.

Apart from in situ deposition, Fe incorporation during the synthesis of cobalt oxide nanowires alters their electronic structure by increasing the average distortion around the cobalt centers and the ratio of Co^2+^ to Co^3+^ in tetrahedral and octahedral sites, respectively.^51^ Furthermore, the reduction of the optimized composite nanowire structure leads to phase segregation and formation of iron oxide nanoparticle-decorated CoO nanowire arrays, as seen in Figure 6a,b.^1^ Reduction of Co_3_O_4_ spinel to CoO rock-salt structure and formation of phase boundaries were found to favor the charge transfer. This led to a significant increase in the current density from 150 to 315 mA/cm^2^ at 1.7 V vs RHE (Figure 6c). *In situ* electrochemical Raman spectroscopy (Figure 6d) and post-structural analyses indicated that the reduced material acted as a precatalyst, which was transformed into an oxyhydroxide species and disordered cobalt oxide spinel during the OER.^1^

The author’s team has also validated that combining boron with cobalt oxide can alter its structure and improve OER performance.^3^ A series of Co–B oxides with variable morphology, crystallinity, and textural parameters were prepared by a facile precipitation method by using NaBH_4_ as reducing agent and B source. Treatment of as-made materials at elevated temperatures induced the formation of crystalline Co_3_O_4_ and amorphous boron oxide. The presence of boron influenced the morphology, crystallization, and surface structure, resulting in a 3-fold increase in the OER activity. Among the different composite oxides, a partially crystalline sample that was calcined at 300 °C exhibited the highest catalytic performance toward the OER by delivering a current density of 235 mA cm^–2^ at 1.7 V_RHE_ and needing an overpotential of 338 mV to reach 10 mA cm^–2^. The alteration of the electrocatalyst and the formation of new species (like CoO_2_ and (oxy)hydroxide) during the OER were found to be highly dependent on the crystallinity of the samples. This finding demonstrates the potential of new compositions to improve energy efficiency and advance the field.

Overall, besides the experimental conditions, various physicochemical properties of catalysts, such as morphology, dimension, size, shape, composition, crystal and electronic structure, surface area, doping, and defects, as well as catalyst and electrode preparation methods can significantly affect their OER activity.^52,53^ Monitoring structural alteration and capturing the surface intermediates and active sites will unravel the mysteries of water electrocatalysis, leading to a better understanding of the process and the development of more active, durable, and affordable catalysts. This would be an absolute game-changer for the future of large-scale green H_2_ production to meet the growing demand.

## 4 Summary and Outlook

Alkaline water electrolysis is a mature technology for green hydrogen production and is receiving more attention for large-scale production. However, there is still a need to optimize the process and develop more affordable, active, and durable electrocatalysts, in particular for the more demanding OER. For catalyst development, it is essential to better understand the process and the reaction mechanism. Atom economy is a key ecological and economical point that should be considered for sustainable catalyst design. It could also be considered to prepare the electrocatalyst working electrode in a single step to reduce the manufacturing cost.

Iron impurities in commercial KOH can activate some electrocatalysts, such as amorphous cobalt oxyhydroxide and Ni-based oxides and Ni, Ag-containing cobalt oxides for the OER. Although this may be advantageous on a small lab scale, it could be problematic for industrial applications, where highly concentrated aqueous KOH solution is used as the electrolyte. Technical and reagent grade KOH have a purity of about 85% and 90%, respectively, and contain other species such as chloride, carbonate, phosphate, sulfate, and organics like formate and acetate, as well as metals like iron and lead. Large deposits of these contaminants, especially carbonate and iron, can block the catalytic sites and lead to catalyst deactivation. It is worthwhile to investigate the effect of impurities in KOH at an AWE pilot plant.

Green hydrogen is shifting our energy landscape and is going to play an essential role in society and a sustainable future. The importance of green hydrogen is well recognized; it is being pushed very rapidly to replace energy vectors and upgrade many industrial processes. However, there is neither sufficient capacity to produce large amounts of green hydrogen nor a suitable infrastructure for its utilization. The cost and accessibility of green hydrogen production are mainly dominated by the price of sustainable electricity and its availability. Further, the fluctuating nature of renewable energy sources is another limiting factor for alkaline water electrolysis plants, which require stationary operating conditions. As a society, we are still not at a point where we can produce our routine electricity from renewable energy sources. Thus, more investment should be made in sustainable electricity production, especially for manufacturing onshore and offshore wind turbines and photovoltaic solar panels. Sustainable electricity will be the biggest bottleneck to large-scale and cheap hydrogen production. It is believed that Africa’s abundant solar and wind energy could make the region a successful producer and global exporter of green hydrogen. However, about 40% and 60% of Africa’s population have no access to electricity and water connection to their homes, respectively. It is very questionable whether the continent’s resources should be exported or rather used for the continent’s development.

After green hydrogen production, its storage, transportation, and safety regulation would pose additional challenges and issues for widespread implementation. The storage of large amounts of hydrogen, either as a gas or as a liquid, is technically and practically challenging. As a gas, it requires very high pressure tanks, and as a liquid, it requires cryogenic temperatures due to its low boiling point of about 20 K. Alternatively, hydrogen can be stored physically in solids or chemically in molecules, such as ammonia. Compared with other hydrogen storage technologies, ammonia synthesis and distribution are well established. However, ammonia decomposition is a very energy intensive process and requires an additional catalytic system for hydrogen generation. Further, high purity hydrogen production is challenging since formation of NO_*x*_ gaseous cannot be avoided with the current technology. There is a need for process optimization and the development of a low temperature ammonia cracking catalyst.

In addition, it is worth noting that economic and infrastructure challenges could also act as barriers to the widespread adoption of green H_2_ in industrial applications. For example, let us consider a steel manufacturing company that wants to switch from using coal as an energy source to using green hydrogen. This switch will require a significant amount of capital and investment to upgrade the necessary infrastructure. Fortunately, some countries, such as Germany, provide financial support for infrastructure upgrades to enable a smooth transition to a more sustainable future.

Overall, it is undeniable that green hydrogen will play a critical role in meeting our future energy needs. However, there are significant challenges that must be overcome to achieve large-scale production, storage, and distribution, as well as integration into the existing energy infrastructure and other sectors such as industry, transportation, and power generation. Nevertheless, hydrogen holds the key to a sustainable future for our society through an energy transition.
